# Response of Spring Diatoms to CO_2_ Availability in the Western North Pacific as Determined by Next-Generation Sequencing

**DOI:** 10.1371/journal.pone.0154291

**Published:** 2016-04-28

**Authors:** Hisashi Endo, Koji Sugie, Takeshi Yoshimura, Koji Suzuki

**Affiliations:** 1 Faculty of Environmental Earth Science/Graduate School of Environmental Science, Hokkaido University, Sapporo, Hokkaido, Japan; 2 CREST, Japan Science and Technology, Sapporo, Hokkaido, Japan; 3 Central Research Institute of Electric Power Industry, Abiko, Chiba, Japan; 4 Research and Development Center for Global Change, Japan Agency for Marine Earth-Science and Technology (JAMSTEC), Yokosuka, Kanagawa, Japan; Mount Allison University, CANADA

## Abstract

Next-generation sequencing (NGS) technologies have enabled us to determine phytoplankton community compositions at high resolution. However, few studies have adopted this approach to assess the responses of natural phytoplankton communities to environmental change. Here, we report the impact of different CO_2_ levels on spring diatoms in the Oyashio region of the western North Pacific as estimated by NGS of the diatom-specific *rbcL* gene (DNA), which encodes the large subunit of RubisCO. We also examined the abundance and composition of *rbcL* transcripts (cDNA) in diatoms to assess their physiological responses to changing CO_2_ levels. A short-term (3-day) incubation experiment was carried out on-deck using surface Oyashio waters under different *p*CO_2_ levels (180, 350, 750, and 1000 μatm) in May 2011. During the incubation, the transcript abundance of the diatom-specific *rbcL* gene decreased with an increase in seawater *p*CO_2_ levels. These results suggest that CO_2_ fixation capacity of diatoms decreased rapidly under elevated CO_2_ levels. In the high CO_2_ treatments (750 and 1000 μatm), diversity of diatom-specific *rbcL* gene and its transcripts decreased relative to the control treatment (350 μatm), as well as contributions of Chaetocerataceae, Thalassiosiraceae, and Fragilariaceae to the total population, but the contributions of Bacillariaceae increased. In the low CO_2_ treatment, contributions of Bacillariaceae also increased together with other eukaryotes. These suggest that changes in CO_2_ levels can alter the community composition of spring diatoms in the Oyashio region. Overall, the NGS technology provided us a deeper understanding of the response of diatoms to changes in CO_2_ levels in terms of their community composition, diversity, and photosynthetic physiology.

## Introduction

Progressive increases in the seawater partial pressure of CO_2_ (*p*CO_2_) and decreases in pH (i.e., ocean acidification, [1]) caused by industrial CO_2_ emissions could affect biological processes in the ocean [2]. Because CO_2_ is the primary substrate for photosynthesis, ocean acidification can enhance CO_2_ availability for marine phytoplankton. Accordingly, ocean acidification could play key roles in controlling productivity and organic matter production [3–5], thereby potentially affecting the biogeochemical and ecological processes in the ocean.

Studies on the impacts of increased CO_2_ levels have been conducted on a variety of phytoplankton species [3, 6, 7, 8]. In the context of the global carbon cycle and feedbacks to climate change, diatoms are an important class of phytoplankton because they are responsible for approximately 40% of total primary production in the global ocean [9]. Comparative studies suggested that the growth of diatoms tended to increase with elevated CO_2_ levels [10, 11]. Indeed, previous field studies have provided some evidence that elevated CO_2_ levels could enhance the growth of diatoms in the Equatorial Pacific [12], the North Atlantic [13], and the Southern Ocean [14]. However, no significant or negative effects of elevated CO_2_ on diatoms have been reported from the bloom-inducing field incubation experiments in the Raunefjord [15], the Western Subarctic Gyre (WSG) of the North Pacific [16], and the Bering Sea basin [17, 18]. These discrepancies could be caused by the species-specific differences in CO_2_ response [19], although experimental conditions and other environmental factors might also affect the outcomes. Hence, detailed taxonomic information on diatoms would be indispensable for understanding their responses to changes in CO_2_ levels in seawater.

In addition, marine diatoms can experience a decrease in CO_2_ availability in some oceanic regions. In the Oyashio region of the western North Pacific, the *p*CO_2_ in surface seawater decrease significantly from winter (ca. 400 μatm) to spring (< 200 μatm) as a consequence of intense diatom blooms [20]. Previous studies have suggested that diatoms can overcome low CO_2_ availability by using biophysical CO_2_-concentrating mechanisms (CCMs), which provide a high CO_2_ concentration at the site of carboxylation [21, 22]. However, some experiments demonstrated that the growth and productivity of diatoms decreased under low CO_2_ (100–220 μatm) conditions [7, 23]. At present, it is largely unknown how diatom assemblages respond to decrease in CO_2_ availability, which occurs during spring blooms in the Oyashio region.

Recently, molecular biological techniques have become important tools for understanding the community composition and biodiversity of natural microbial assemblages including phytoplankton based on DNA sequence information [24–26]. It is reported that the abundance of particular genes can be an indicator of phytoplankton pigment biomass [27]. These techniques can also be applied to assess the responses of phytoplankton assemblages under different CO_2_ levels [28–30]. For example, Hopkinson et al. [28] used 18S rRNA and nitrate reductase (NR) gene fragments to estimate the community compositions of haptophytes and diatoms, respectively, in different CO_2_ levels. In addition, the *rbcL* gene, which encodes the large subunit of the enzyme ribulose bisphosphate carboxylase/oxygenase (RubisCO), was also used to estimate the abundance and community composition of phytoplankton assemblages during the CO_2_-controlled mesocosm experiment conducted off the Norwegian coast [29]. These authors demonstrated a rapid shift in the community composition of pico-sized prasinophytes (i.e. *Bathycoccus* and *Micromonas*), which were difficult to identify by light microscopy, in response to increased CO_2_ levels.

In addition, mRNA (cDNA) transcripts of functional genes could be a proxy for physiological responses of phytoplankton assemblages to environmental change. For example, the transcript levels of the *rbcL* gene could be an indicator of productivity because CO_2_ is assimilated via RubisCO in the Calvin-Benson-Bassham (CBB) cycle [31, 32]. Changes in gene expression and protein synthesis levels of RubisCO occur within a few hours in response to environmental change such as light and nutrient availability [33–35]. Therefore, it can be used to infer the potential effect of different CO_2_ levels on the natural phytoplankton assemblages even from short-term incubation experiment. Endo et al. [27] showed that diatom-specific *rbcL* transcripts decreased in response to elevated CO_2_ levels after 2 or 3 days of incubation in the Bering Sea. They also showed that a shift occurred in the community composition of photosynthetically active diatoms with an increase in seawater CO_2_ levels. However, the conventional molecular cloning method with Sanger sequencing [36], which was used in previous studies, is sometimes inadequate for the extraction of comprehensive information from environmental samples, due to limited throughput, and may therefore underestimate the taxonomic richness of microbial assemblages [37, 38].

Recent advances in next-generation sequencing (NGS) technologies can overcome this limitation because these deep sequencing technologies can now generate several hundred thousand reads per sample [39]. Practically, metagenomic and amplicon sequencing using NGS platforms have been used to reveal the community composition and/or diversity of bacteria [40], phytoplankton [41], and zooplankton [42] in marine environments. However, to the best of our knowledge, these new technologies have not been used to estimate the effects of CO_2_ availability on marine phytoplankton assemblages.

Here, we report the use of NGS technologies in combination with real-time PCR (qPCR) and HPLC pigment analysis to improve our understanding of the effects of CO_2_ availability on the community structure and photosynthetic physiology of diatoms in the Oyashio region. Because of the massive diatom blooms in spring, this region has one of the greatest capacities for seasonal biological drawdown of *p*CO_2_ in surface waters among the world’s oceans [43]. Field observations revealed that the blooms were mainly composed of centric diatoms, such as *Chaetoceros* spp. and *Thalassiosira* spp. [44–47]. Taniguchi [48] noted that these diatoms contribute to the efficient energy transfer to higher trophic levels in the Oyashio region from spring to summer. Consequently, the spring diatom blooms significantly contribute to the formation of the highly productive fishing grounds in Oyashio and its surrounding waters [48, 49]. However, despite the crucial roles of diatom blooms in the ecosystems and biogeochemical processes, there are no reports on the impacts of different CO_2_ availability on phytoplankton assemblages in the Oyashio region. We therefore conducted an on-deck incubation experiment using spring Oyashio waters under different *p*CO_2_ levels.

## Materials and Methods

Seawater samples used in this study were not collected in a protected area. No specific permissions were required for sampling of seawater in the locality. The samples taken for our study did not involve endangered or protected species.

### 2.1. Experimental setup and sampling

**Fig 1:**
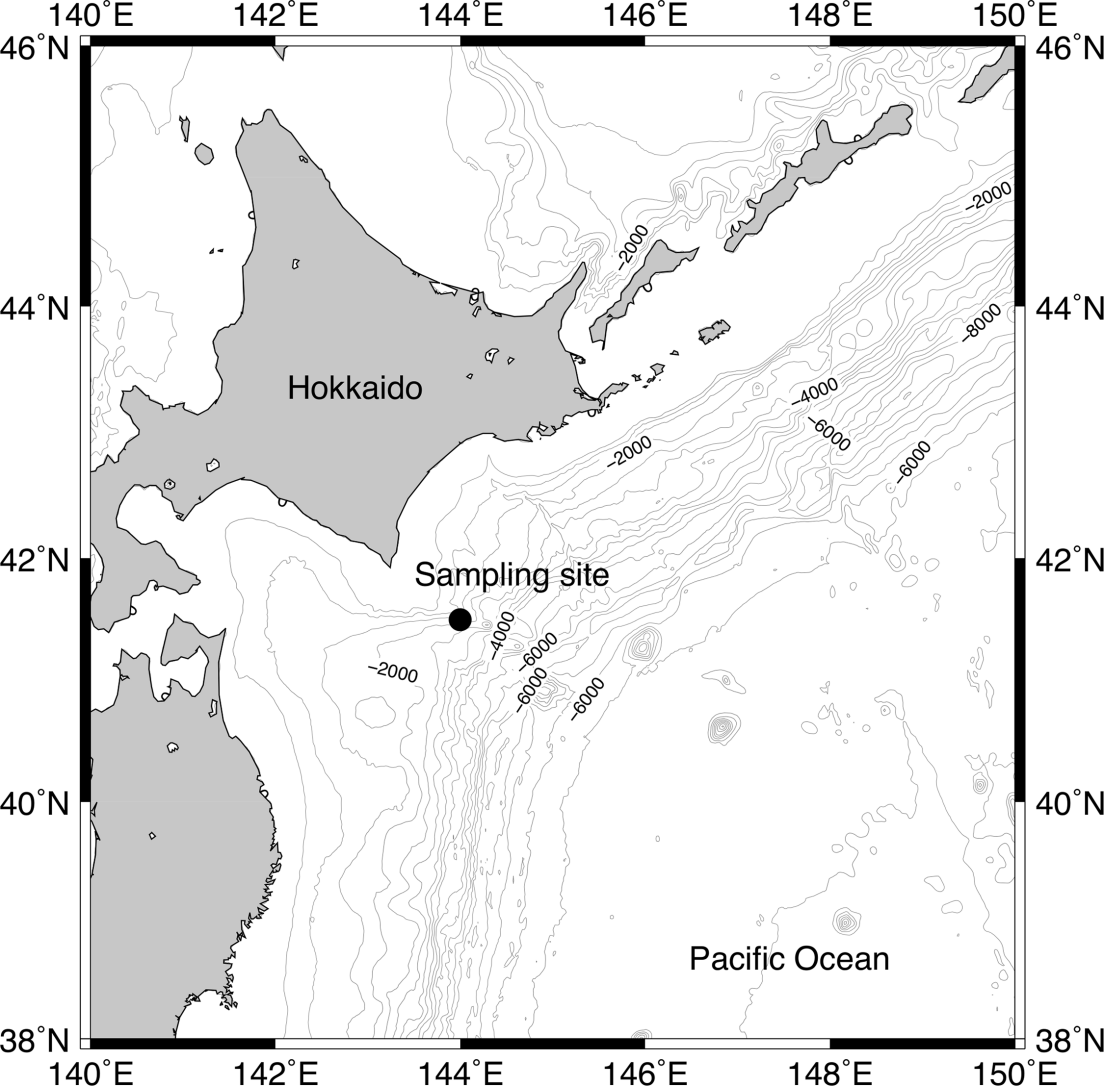
Location of the sampling station (41° 30’ N, 144° 00’ W) for the incubation experiment.

The study was carried out aboard the R/V *Tansei Maru* (JAMSTEC) during the KT-11-7 cruise in May 2011. Water samples were collected from 10-m depth at a station (41° 30’ N, 144° 00’ E, 1668 m depth, Fig 1) in the Oyashio region of the western North Pacific on 9 May with Niskin-X bottles attached to a CTD-CMS system. A total of 200 L of seawater was poured into four 50 L polypropylene carboys through silicon tubing with 197 μm mesh Teflon nets to remove large particles. Prior to incubation, FeCl_3_ solution (5 nmol L^−1^ in final concentration) was added to the carboys to promote the development of phytoplankton blooms because the growth of bloom-forming diatoms can be limited by low iron (Fe) availability in this region [45, 46]. Subsamples were taken from each carboy and poured into triplicate acid-cleaned 12-L polycarbonate bottles (12 bottles total) for incubation. Initial (day 0) samples were collected from each carboy. Initial seawater *p*CO_2_ (i.e., 350 μatm) was measured using the Non-Dispersive Infra-Red (NDIR) method using a portable CO_2_ analyzer (CO2-09, Kimoto Electric), and the resulting value was used as the control benchmark. To control *p*CO_2_ in the incubation bottles, air mixtures containing 180, 350, 750, and 1000 μatm CO_2_ were bubbled into the incubation bottles. The flow rate of the gases was set at 100 mL min^–1^ for the first 24 h, and thereafter maintained at 50 mL min^–1^. Incubation was conducted on deck in a tank with running surface seawater for 3 days, maintaining *in situ* temperature of 5°C and 50% surface irradiance, which was adjusted using a neutral density screen. The temperature of the incubation tanks ranged between 4.7 and 6.0°C throughout the incubation. All of the samples, except on day 0, were collected between 5:00 and 6:00 a.m.

### 2.2. Carbonate chemistry, nutrients, and Chl *a* analyses

Samples for total alkalinity (TA), dissolved inorganic carbon (DIC), nutrients, and size-fractionated chlorophyll *a* (Chl *a*) were collected on days 0, 0.6, 1.6, and 2.6 (hereafter days 0, 1, 2, and 3, respectively) from the incubation bottles. Samples for TA and DIC were collected in airtight glass vials and poisoned with HgCl_2_ prior to their storage at 4°C for analysis on shore. Concentrations of TA and DIC were measured with a total alkalinity analyzer using the potentiometric Gran Plot method (ATT-05, Kimoto Electric) following Edmond [50]. The values of *p*CO_2_ and pH were calculated from the TA and DIC using the CO2SYS program [51]. Nutrient samples were poured into 10 mL plastic tubes and stored at −20°C until they were analyzed on shore. Concentrations of nitrate plus nitrite, nitrite, phosphate, and silicic acid were measured using a QuAATro-2 continuous-flow analyzer (Gran+Luebbe). Size-fractionated Chl *a* samples were collected onto a 10 μm pore size polycarbonate filter without vacuum or onto Whatman GF/F filters under a gentle vacuum (< 0.013 MPa). Pigments were extracted in *N*,*N*-dimethylformamide (DMF; [52]), and Chl *a* concentrations were then determined using a Turner Design fluorometer (model 10-AU) with the non-acidification method of Welschmeyer [53].

### 2.3. FIRe fluorometry

To obtain the maximum photochemical quantum efficiency (*F*_v_/*F*_m_) of photosystem II (PSII) for phytoplankton in each bottle, a Fluorescence Induction and Relaxation (FIRe) fluorometer (Satlantic) was used. The water samples were collected daily from the incubation bottles and were measured following Sugie et al. [18].

### 2.4. HPLC and CHEMTAX analyses

Pigment samples for high-performance liquid chromatography (HPLC) were collected on days 0, 2, and 3. The water samples (600–1000 mL) were filtered onto Whatman GF/F filters under a gentle vacuum (< 0.013 MPa), flash-frozen in liquid nitrogen and then stored in a deep freezer (−80°C) until analysis. HPLC pigment analysis was conducted following the method of Endo et al. [16]. The net growth rate (μ, day^−1^) of each pigment was calculated from the following equation:

$$\mu =\frac{dC}{dt}\left(\frac{1}{C}\right)$$

where *C* is the pigment concentration (μg L^−1^) and *t* is incubation time (i.e., 2.6 days). Based on the chemotaxonomic pigment concentrations, phytoplankton community structure was estimated using the CHEMTAX program [54] following Endo et al. [16]. The initial and final matrices of pigment:Chl *a* ratios are shown in S1 and S2 Tables, respectively.

### 2.5. qPCR and qRT-PCR

Samples for DNA and RNA analyses were collected on days 0, 2 and 3. DNA samples (300–400 mL) were collected onto 0.2 μm pore size polycarbonate Nuclepore membrane filters (Whatman) with gentle vacuum (< 0.013 MPa), flash frozen in liquid nitrogen and then stored in a deep freezer at −80°C until analysis. Seawater samples (300–400 mL) for RNA analysis were filtered onto 0.2 μm pore size polycarbonate Nuclepore filters (Whatman) with gentle vacuum (< 0.013 MPa) and then stored in 1.5-mL cryotubes previously filled with 0.2 g of muffled 0.1-mm glass beads and 600 μL of RLT buffer (Qiagen) with 10 μL mL^−1^ β-mercaptoethanol (Sigma-Aldrich). The RNA samples were flash-frozen in liquid nitrogen and stored in a deep freezer at −80°C until analysis. The detailed methodologies of total DNA and RNA extractions were described in Endo et al. [16] and Endo et al. [27], respectively. The extracted RNA was reverse-transcribed into cDNA using the PrimeScript RT Reagent Kit with gDNA Eraser (Takara). To quantify the diatom-specific *rbcL* gene (DNA) and its transcripts (cDNA), qPCR and qRT-PCR were performed according to the method of Endo et al. [27].

### 2.6. Ion Torrent Personal Genome Machine (PGM) sequencing

Gene fragments of diatom-specific *rbcL* sequences were amplified from the extracted DNA or cDNA samples with barcoded fusion primer pairs, which included the primer set designed by John et al. [55]: forward primer, 5’-GATGATGARAAYATTAACTC-3’; reverse primer, 5’-TAWGAACCTTTWACTTCWCC-3’. The forward primer included the A-adaptor (5’-CCATCTCATCCCTGCGTGTCTCCGAC-3’), key (5’-TCAG-3’) and multiplex identifier (MID) sequences set by the manufacturer (Life Technologies), whereas the reverse primer included the truncated Pi-adapter (trP1: 5’-CCTCTCTATGGGCAGTCGGTGAT-3’) sequence. Triplicate PCR amplifications were conducted for each samples using the TaKaRa Ex *Taq* Hot Start Version (Takara). The thermal cycling conditions were 60 s at 94°C for initial denaturation, then 30 cycles consisting of 10 s at 98°C, 30 s at 52°C, and 60 s at 72°C, followed by 10 min at 72°C for final extension. The PCR amplification was checked with 1.5% agarose gel electrophoresis. Amplicons were purified using AMPure beads (Beckman Coulter) and then quantified by an Agilent 2100 Bioanalyzer using the DNA 1000 Kit (Agilent Technologies) following the manufacturer’s instructions. Then, the PCR templates were diluted to the final concentration of 26 pmol L^−1^, and these were mixed equally in terms of concentration. Emulsion PCR was performed using an Ion One Touch 2 system with Ion PGM Template OT2 200 kit (Life Technologies) according to the manufacturer’s protocol. The emulsion PCR products were enriched using an Ion One Touch ES (Life Technologies) and then loaded onto an Ion 318 v2 chip (Life Technologies). Sequencing of the amplicon libraries was performed using an Ion Torrent PGM system with the Ion PGM sequencing 200 kit v2 (Life Technologies) according to the manufacturer’s protocol.

### 2.7. Sequence analyses

#### 2.7.1. Quality filtering

Sequences with low quality, polyclonal sequences, and sequences that did not match against the A-adapter were initially filtered out with the Torrent Suite Software (Life Technologies), and the data so obtained were exported as FASTQ files. The complete run files in each sample were deposited in the DDBJ Sequence Read Archive (DRA) with the accession number DRA003722. Additional quality controls were performed using the software FASTX-Toolkit (http://hannonlab.cshl.edu/fastx_toolkit/). Sequencing reads were excluded if they met any of the following criteria: (i) reads not containing the trP1 adapter sequence; (ii) reads not matching the reverse primer sequence; (iii) reads shorter than 112 bp; and (iv) reads with average quality score below 25. Sequence quality statistics of raw and quality-filtered libraries are shown in S3 Table.

#### 2.7.2. Taxonomic assignment

For taxonomic classification, contig assembly was achieved using the software SeqMan NGen (DNASTAR). Fifty thousand reads for each library were assembled into contigs with ≥ 97% sequence identity. The representative sequences of each contig containing with ≥ 100 reads (≥ 0.2% for total reads) were compared with *rbcL* sequences deposited in GenBank database (http://www.ncbi.nlm.nih.gov) using the BLAST query engine. Contigs that have ≥ 93% sequence similarity with known *rbcL* sequences were classified into the following groups: Chaetocerotaceae, Coscinodiscaceae, Cymatosiraceae, Rhizosoleniaceae, Stephanodiscaceae, Thalassiosiraceae, Achananthaceae, Bacillariaceae, Naviculaceae, Fragilariaceae, unidentified diatoms, and other eukaryotes. Contigs related to two or more diatom families with the same sequence similarity were assigned as unidentified diatoms. Other eukaryotes consisted of the contigs that were closely related to the sequences from organisms other than diatoms deposited in GenBank. A phylogenetic tree constructed with *rbcL* reference sequences is shown in S4 Fig.

#### 2.7.3. Sequence-based statistical analyses

Principal Component Analysis (PCA) was used to recognize the relationships among *p*CO_2_ levels in the DNA or cDNA libraries by reducing the multivariate information using the software R (http://www.r-project.org/). The ordination was performed based on the matrix of relative compositions at the family level in the libraries. In addition, ten thousand reads in each library were extracted for diversity analyses by the software mothur v. 1.25.0 [56]. All libraries were merged and clustered into operational taxonomic units (OTUs) with ≥ 97% sequence similarity cutoff. Singleton OTUs were then removed from the libraries. Genetic diversity was assessed based on the number of OTUs, Shannon-Wiener index (*H*’, [57]), and Simpson’s index (1-*D*, [58]).

### 2.8. Significance test

Statistical analyses were performed with the software R (http://www.r-project.org). To evaluate the statistically significant differences among CO_2_ treatments, Kruskal-Wallis one-way analysis of variance (ANOVA) was used. Holm’s test for multiple comparisons was also used to identify the source of variance. For all tests, *p* < 0.05 was considered significant.

## Results

### 3.1. Carbonate chemistry and nutrients

The initial concentrations of TA and DIC in the seawater were 2239.4 and 2074.7 μmol kg^–1^, respectively (Table 1). Initial values of calculated pH and *p*CO_2_ were 8.1 and 333.1 μatm, respectively (Table 1). Our incubation system successfully created significant gradients in *p*CO_2_ and pH (S1 Fig) in seawater without any variation in TA between CO_2_ treatments. Significant gradients in *p*CO_2_ were achieved within 0.6 days, and it took at least 1.5 days to reach the intended *p*CO_2_ values (S1A Fig). The mean *p*CO_2_ values after day 2 in the 180, 350, 750, and 1000 μatm CO_2_ treatments were 232, 328, 628, and 822 μatm, respectively. The initial concentrations of nitrate, phosphate, and silicic acid in seawater were 13.72, 0.99, and 11.76 μmol L^–1^, respectively, and the macronutrients remained until the end of the incubation (Table 1).

**Table 1:** Carbonate chemistry and nutrient concentrations (value ± 1 SD, n = 3, except for the initial: n = 4) at the initial (day 0) or end (day 3) of the incubation.

	TA	DIC	*p*CO_2_	pH	Nitrate	Phosphate	Silicic acid
	(μmol kg^−1^)	(μmol kg^−1^)	(μatm)	(Total scale)	(μmol L^−1^)	(μmol L^−1^)	(μmol L^−1^)
Initial	2239.4 ± 1.2	2074.7 ± 0.9	333.1 ± 1.9	8.10 ± 0.00	13.72 ± 0.04	0.99 ± 0.03	11.76 ± 0.05
180 μatm	2245.3 ± 0.4	2014.9 ± 2.0	225.6 ± 2.9	8.25 ± 0.00	13.22 ± 0.04	0.99 ± 0.01	11.06 ± 0.04
350 μatm	2243.6 ± 2.3	2078.5 ± 1.2	333.2 ± 3.6	8.10 ± 0.00	13.13 ± 0.02	1.00 ± 0.00	10.99 ± 0.06
750 μatm	2245.6 ± 1.2	2172.2 ± 0.8	645.6 ± 1.6	7.84 ± 0.00	13.26 ± 0.01	1.02 ± 0.00	11.27 ± 0.07
1000 μatm	2245.0 ± 0.7	2202.9 ± 2.7	839.9 ± 15.8	7.73 ± 0.01	13.28 ± 0.01	1.01 ± 0.01	11.19 ± 0.06

### 3.2. Chl *a* and *F*_v_/*F*_m_

The initial concentration of Chl *a* was 0.70 μg L^–1^ (≥ 10 μm: 0.18 μg L^–1^, GF/F (ca. 0.7 μm) −10 μm: 0.52 μg L^–1^). Concentrations of Chl *a* increased over time and reached a maximum at the end of the incubation (Table 2, S2 Fig). Although there was no significant difference in Chl *a* concentration in the small (GF/F−10 μm) fraction among samples incubated at different CO_2_ levels during the sampling period, the Chl *a* concentration in the large (≥10 μm) fraction was significantly lower in the high (750 and 1000 μatm) CO_2_ treatments than in the control (350 μatm) treatment on day 3 (Kruskal-Wallis ANOVA, Holm’s test, *p* < 0.05).

**Table 2:** Size-fractionated Chl *a* and *F*_v_/*F*_m_ parameters (value ± 1 SD, n = 3, except for the initial: n = 4) at the initial (day 0) or end (day 3) of the incubation.

	Small-sized Chl *a*	Large-sized Chl *a*	*F*_v_*/F*_m_
	(μg L^−1^)	(μg L^−1^)	(Relative)
Initial	0.52 ± 0.03	0.18 ± 0.04	0.38 ± 0.01
180 μatm	0.89 ± 0.01	0.60 ± 0.08	0.43 ± 0.01
350 μatm	0.87 ± 0.01	0.70 ± 0.02	0.44 ± 0.01
750 μatm	0.85 ± 0.02	0.49 ± 0.02	0.41 ± 0.01
1000 μatm	0.87 ± 0.00	0.51 ± 0.03	0.41 ± 0.01

Initial values of *F*_v_/*F*_m_ were ca. 0.38 (Table 2, S3 Fig). The values of *F*_v_/*F*_m_ increased to ca. 0.44 for all treatments on day 1 and remained above 0.41 until the end of the incubation. The *F*_v_/*F*_m_ values of samples incubated under different CO_2_ levels were not significantly different (Kruskal-Wallis ANOVA, *p* > 0.05).

### 3.3. Phytoplankton pigments

**Fig 2:**
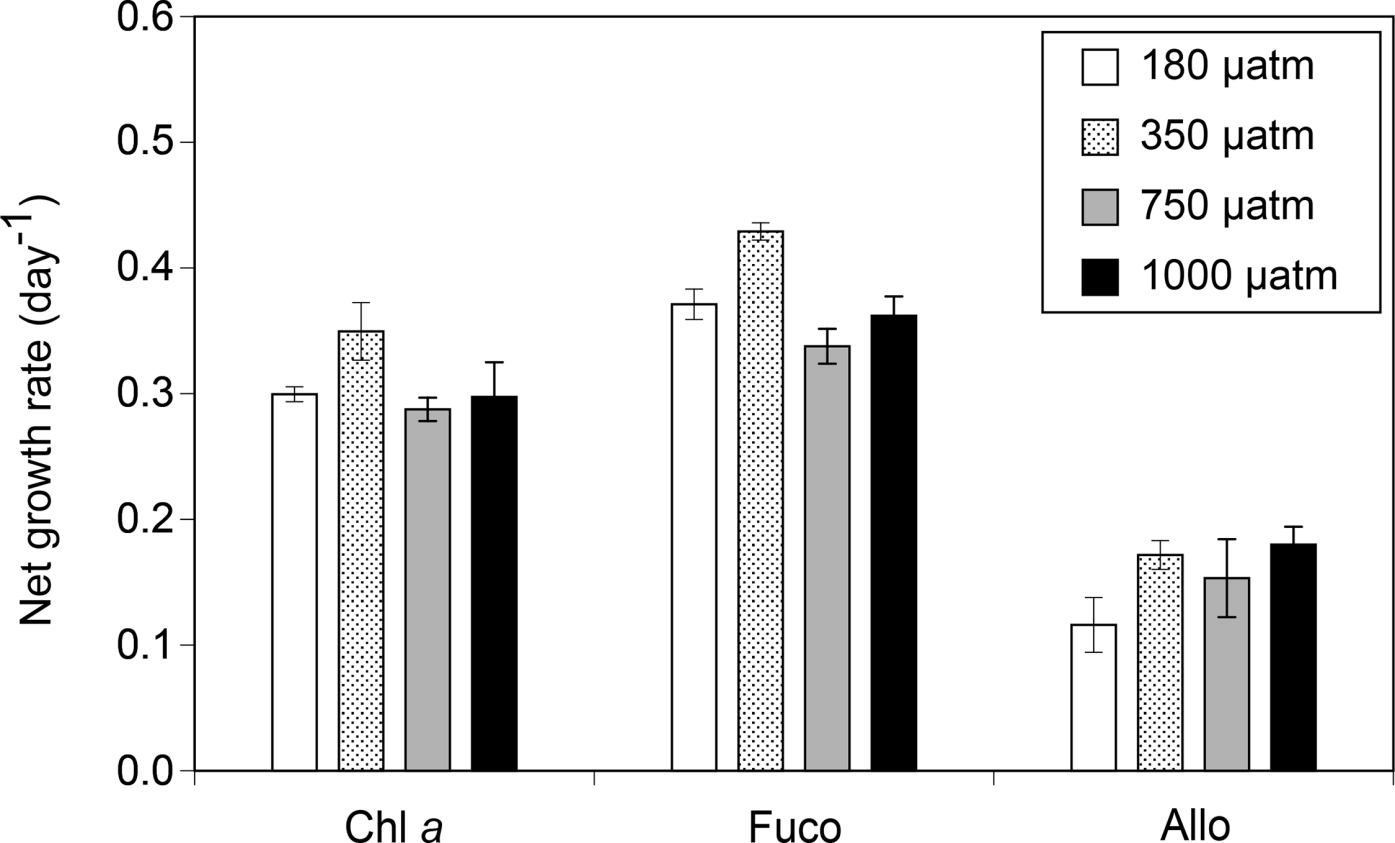
Net growth rates of chlorophyll *a* (Chl *a*), fucoxanthin (Fuco), and alloxanthin (Allo) concentrations as estimated by HPLC during the incubation. Error bars denote ± 1 SD (n = 3).

In the initial seawater, the concentrations of fucoxanthin (Fuco) and alloxanthin (Allo), which are biomarkers of diatoms in the Oyashio region during spring [47] and of cryptophytes [59], respectively, were relatively high among the carotenoids detected in this study. During incubation, the net growth rates of Fuco were the highest among all of the chemotaxonomic pigments in all of the incubation bottles (Fig 2). Significant differences in the net growth rates of Fuco were found among the CO_2_ treatments (Kruskal-Wallis ANOVA, *p* < 0.05), and the value for the control treatment was higher than that for the other treatments (Holm’s test, *p* < 0.05) (Fig 2). The net growth rates of Chl *a* and Allo were not significantly different among the CO_2_ treatments (Kruskal-Wallis ANOVA, *p* > 0.05).

**Fig 3:**
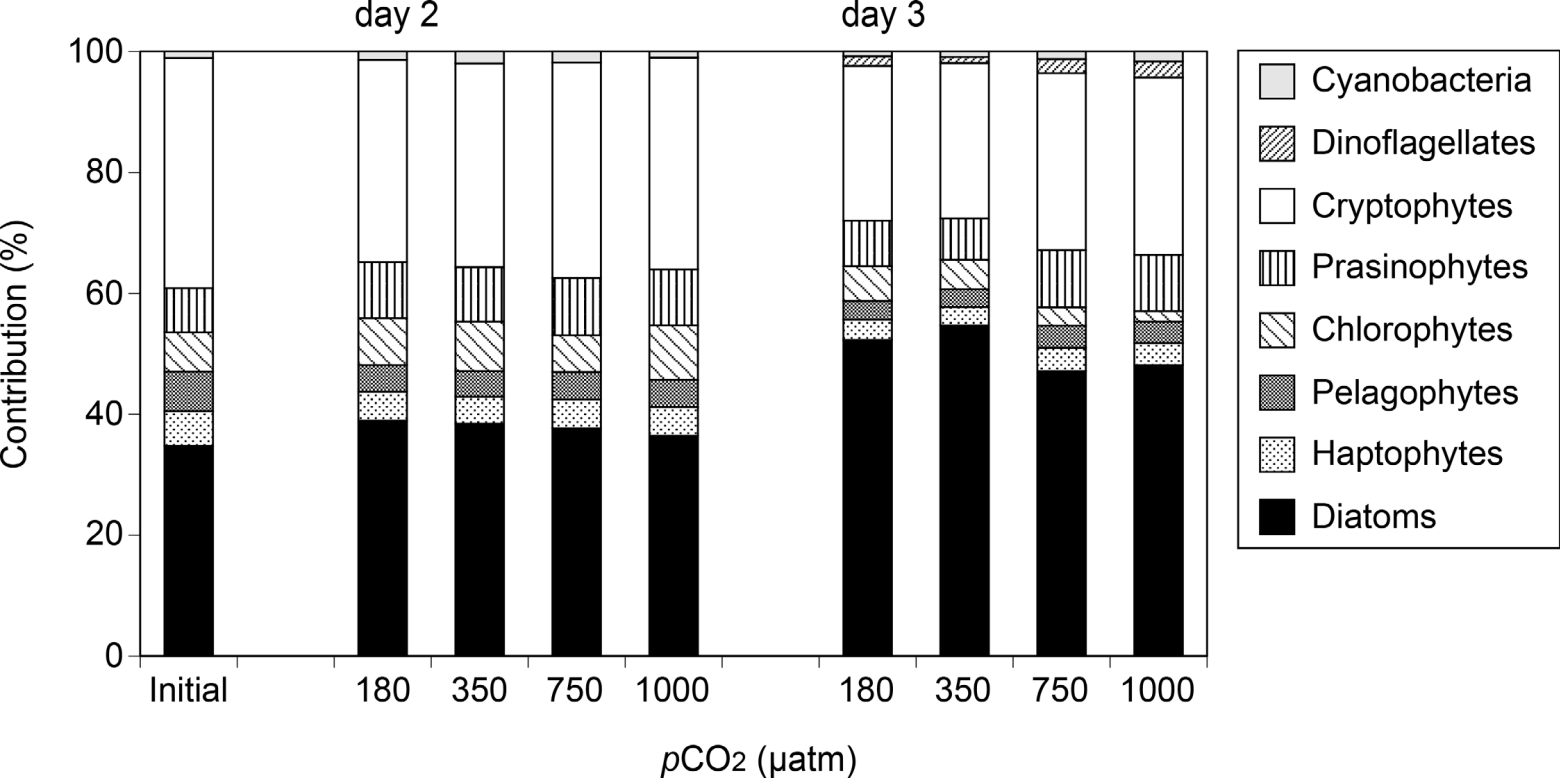
Mean contributions of each phytoplankton group to total Chl *a* biomass as estimated by CHEMTAX. Error bars denote ± 1 SD (n = 3).

The initial phytoplankton community was mainly dominated by cryptophytes and diatoms (38.1% and 34.8% contributions to the Chl *a* biomass, respectively) (Fig 3). Contributions of diatoms to the Chl *a* biomass increased (> 47% in all CO_2_ treatments on day 3), whereas those of cryptophytes decreased (< 30%). The diatom contributions in the high-CO_2_ treatments are significantly lower than those in the low or control CO_2_ treatments on day 3 (Kruskal-Wallis ANOVA, Holm’s test, 180 and 350 μatm > 750 and 1000 μatm, *p* < 0.05). On the other hand, contributions of cryptophytes, haptophytes, and pelagophytes to Chl *a* biomass were significantly higher in the high-CO_2_ treatments on day 3 (Kruskal-Wallis ANOVA, Holm’s test, 180 and 350 μatm < 750 and 1000 μatm, *p* < 0.05).

### 3.4. Copy numbers of *rbcL* DNA and cDNA of diatoms

**Fig 4:**
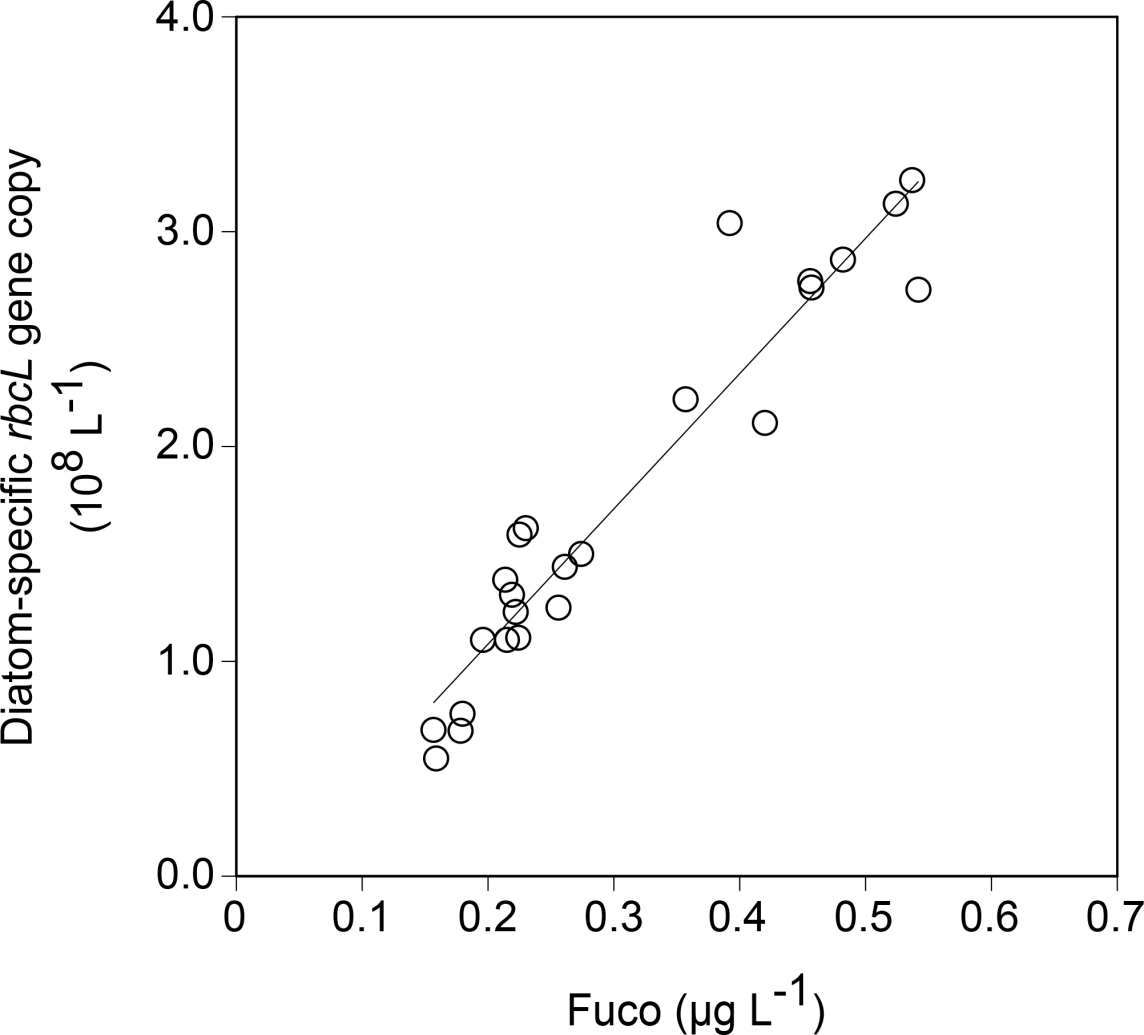
Relationship between fucoxanthin (Fuco) concentration and diatom-specific *rbcL* copy number (y = 6.29×10^8^x − 1.90×10^8^, *R*^2^ = 0.910, *p* < 0.001, n = 25).

**Fig 5:**
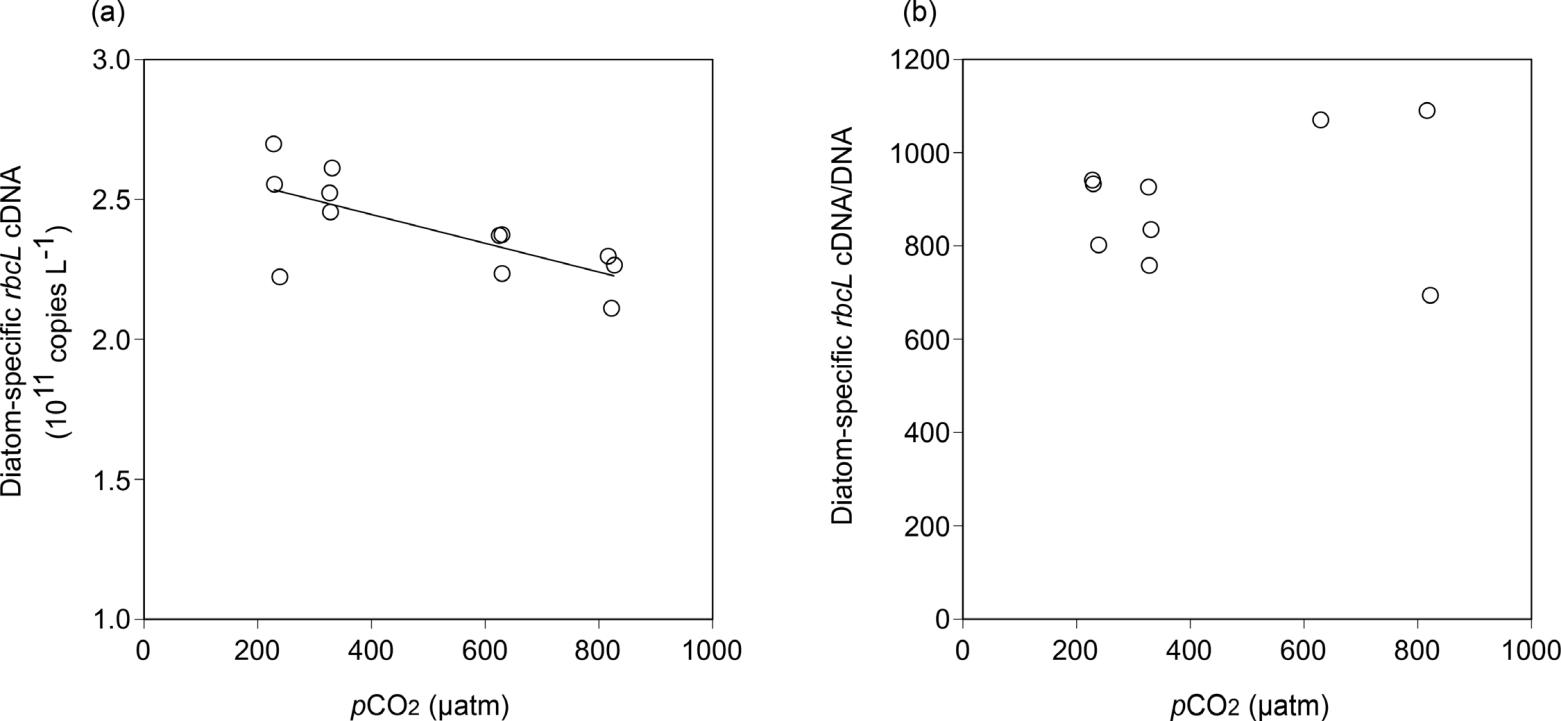
Relationships between seawater *p*CO_2_ (calculated values from TA and DIC) and (a) diatom-specific *rbcL* cDNA copies and (b) diatom-specific *rbcL* cDNA copies normalized to *rbcL* gene copy on day 3.

A significant positive correlation between Fuco concentration and the diatom-specific *rbcL* gene copy count was found during the incubation (*R*^2^ = 0.910, *p* < 0.001, *n* = 25) (Fig 4). In addition, a significant negative correlation was found between seawater *p*CO_2_ and diatom-specific *rbcL* cDNA copy count on day 3 (*R*^2^ = 0.506, *p* < 0.01, *n* = 12) (Fig 5A). However, no significant correlation existed between the seawater *p*CO_2_ and the diatom-specific *rbcL* cDNA abundance normalized to the DNA abundance (cDNA/DNA) on day 3 (*R*^2^ = 0.035, *p* > 0.05, *n* = 9) (Fig 5B).

### 3.5. Molecular OTU-based analyses of diatom-specific *rbcL* DNA and cDNA

The sequence data ranged from 340,809 to 1,194,481 reads per sample were generated from the Torrent Suite software, and approximately 80% of the raw sequences were removed by the subsequent procedures (S3 Table). As a result of the data filtering, average quality score of our sequence data increased from ca. 27.7 to ca. 31.4, indicating that the average error rate of our libraries was below 0.1%. The rarefaction curves plateaued when 10,000 sequences were calculated for each treatment (S5 Fig). The NGS libraries of diatom-specific *rbcL* DNA and cDNA from the initial sample contain 117 and 113 OTUs, respectively (Table 3). On day 0, genetic diversity in the DNA library, calculated using the Shannon and Simpson indices, was 2.30 and 0.772, for *H*’ and 1-*D*, respectively. Similarly, those in the cDNA library were 2.50 and 0.829, respectively. At the end of the incubation, the highest OTU values and diversity indices were observed in the control treatment in the DNA and cDNA libraries (Table 3). The values of *H*’ and 1-*D* were significantly higher in the 180 and 350 μatm CO_2_ treatments than in the 750 and 1000 μatm CO_2_ treatments on day 3 (*t*-test, *p* < 0.05).

**Table 3:** Number of OTUs and diversity indices (value ± 95% confidence interval) for *rbcL* DNA and cDNA libraries obtained from the initial sample and the incubation bottles on day 3.

	DNA			cDNA		
	OTUs	*H*'	1-*D*	OTUs	*H*'	1-*D*
Initial	117	2.30 ± 0.04	0.772 ± 0.007	113	2.50 ± 0.03	0.829 ± 0.005
180 μatm	95	2.22 ± 0.03	0.753 ± 0.008	95	2.35 ± 0.03	0.808 ± 0.006
350 μatm	114	2.27 ± 0.03	0.759 ± 0.008	103	2.35 ± 0.03	0.809 ± 0.006
750 μatm	103	2.15 ± 0.03	0.740 ± 0.007	95	2.28 ± 0.03	0.785 ± 0.005
1000 μatm	101	2.16 ± 0.03	0.745 ± 0.007	98	2.24 ± 0.03	0.774 ± 0.005

**Fig 6:**
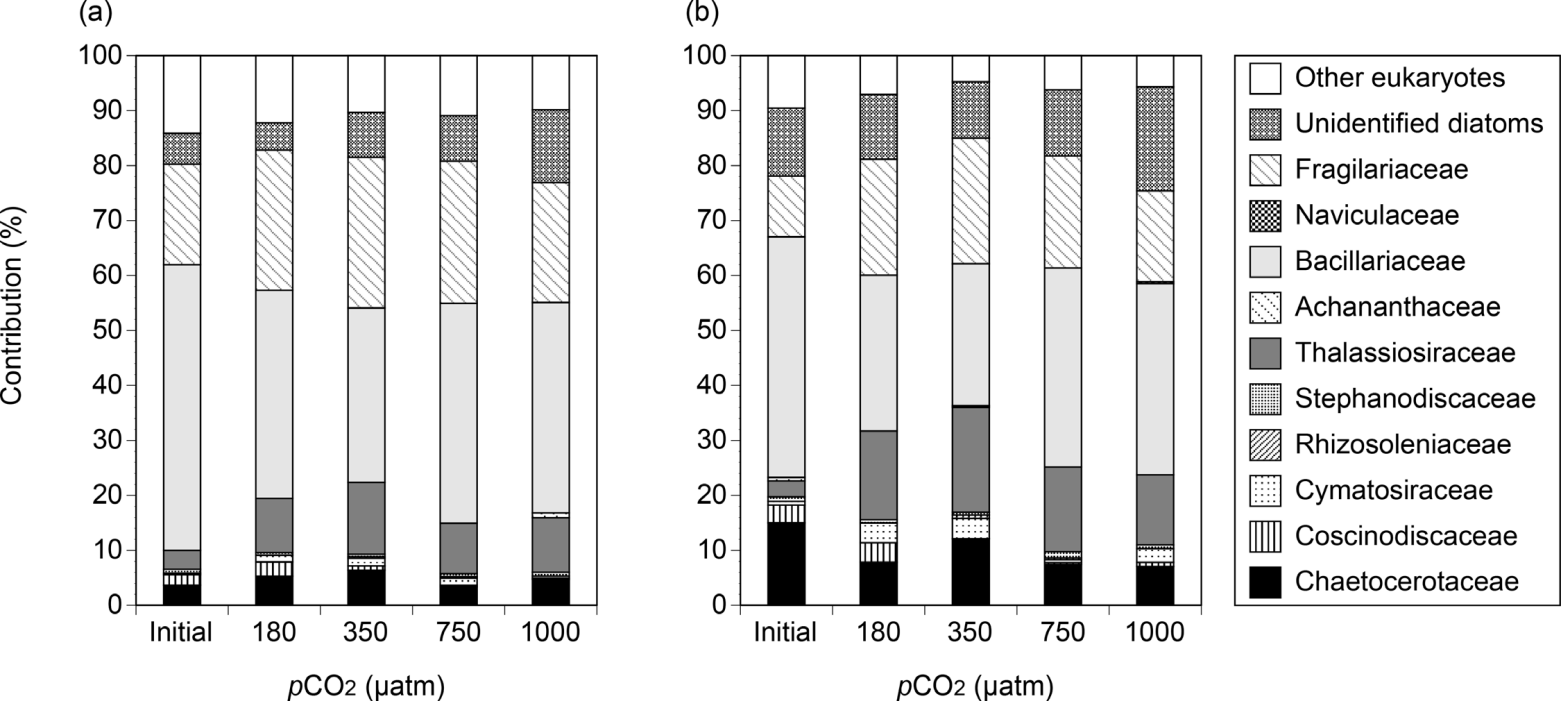
Relative taxonomic contributions (%) in the *rbcL* (a) DNA and (b) cDNA libraries obtained from the initial samples and the 180, 350, 750, and 100 μatm CO_2_ treatments on day 3.

**Fig 7:**
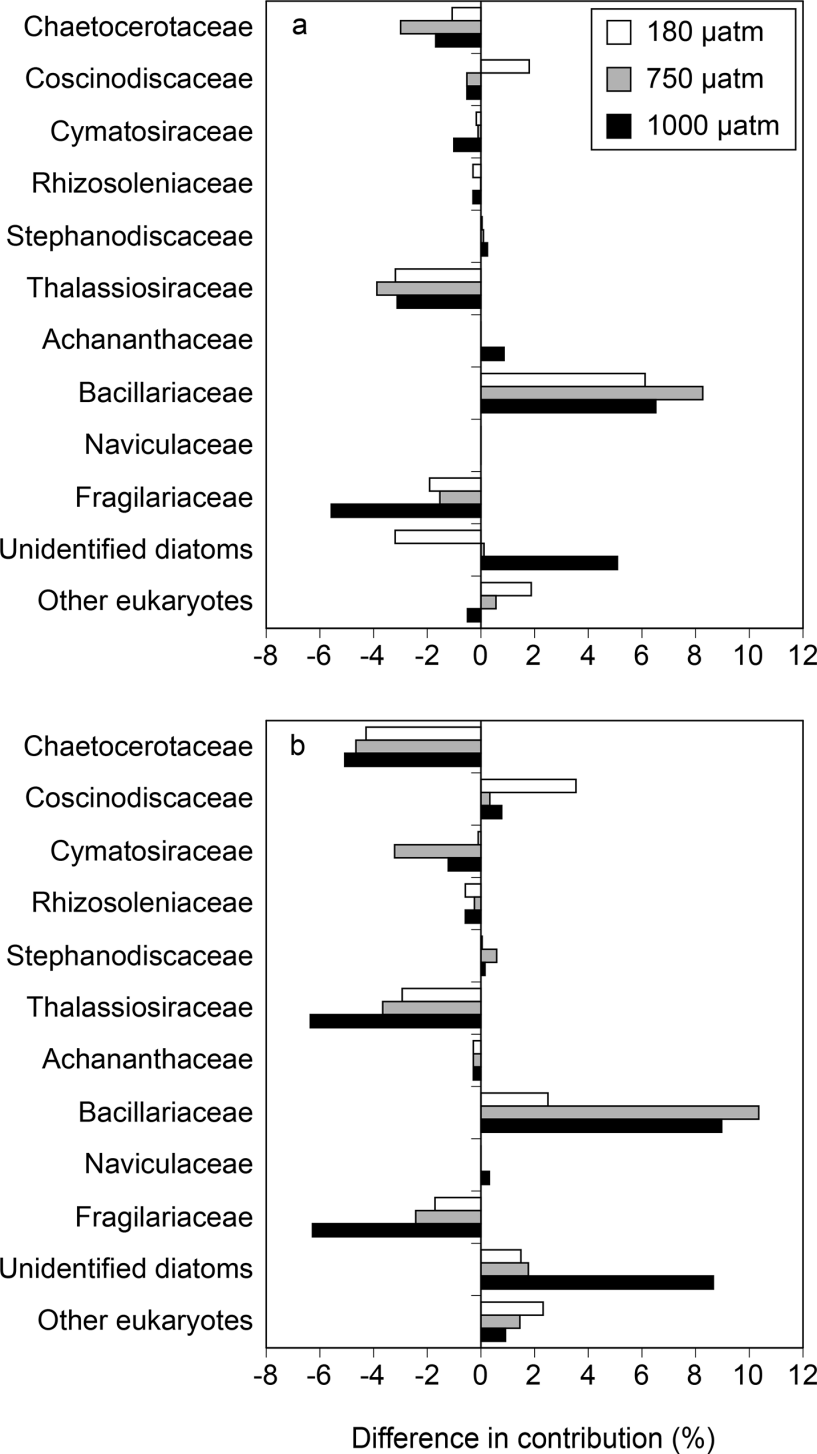
Percent differences in *rbcL* contribution (%) between the control (350 μatm CO_2_) and the other *p*CO_2_ treatments in the (a) DNA and (b) cDNA libraries.

For the DNA and cDNA libraries, more than 85% of the sequences were closely related to the known diatom sequences with ≥ 93% sequence similarity. In the initial seawater, the dominant diatom groups in the *rbcL* DNA were Bacillariaceae and Fragilariaceae (52.0% and 18.3%, respectively), followed by unidentified diatoms, Chaetocerotaceae, Thalassiosiraceae, and Coscinodiscaceae (5.6%, 3.7%, 3.4%, and 1.9%, respectively) (Fig 6A). Initial *rbcL* cDNA consisted mainly of Bacillariaceae and Chaetocerotaceae (43.8% and 15.0%, respectively), followed by unidentified diatoms, Fragilariaceae, Coscinodiscaseae and Thalassiosiraceae (12.4%, 11.0%, 3.2% and 2.9%, respectively) (Fig 6B). At the end of the incubation, the contributions of Thalassiosiraceae and Fragilariaceae to the total population increased relative to the initial contribution for all of the CO_2_ treatments in the DNA and cDNA libraries (Fig 6). The contributions of Bacillariaceae to the total became higher in the 180, 750 and 1000 μatm CO_2_ treatments compared with the control (Fig 7A and 7B). On the other hand, the contributions of Fragilariaceae, Thalassiosiraceae, and Chaetocerotaceae to the total decreased in the 180, 750 and 1000 μatm CO_2_ treatments relative to the control on day 3. Percent differences in Bacillariaceae, Fragilariaceae, Thalassiosiraceae, and Chaetocerotaceae were higher in the cDNA than in the DNA. The contributions of Coscinodiscaceae to the total population increased in the low CO_2_ treatment relative to the control in both the DNA and cDNA libraries on day 3.

**Fig 8:**
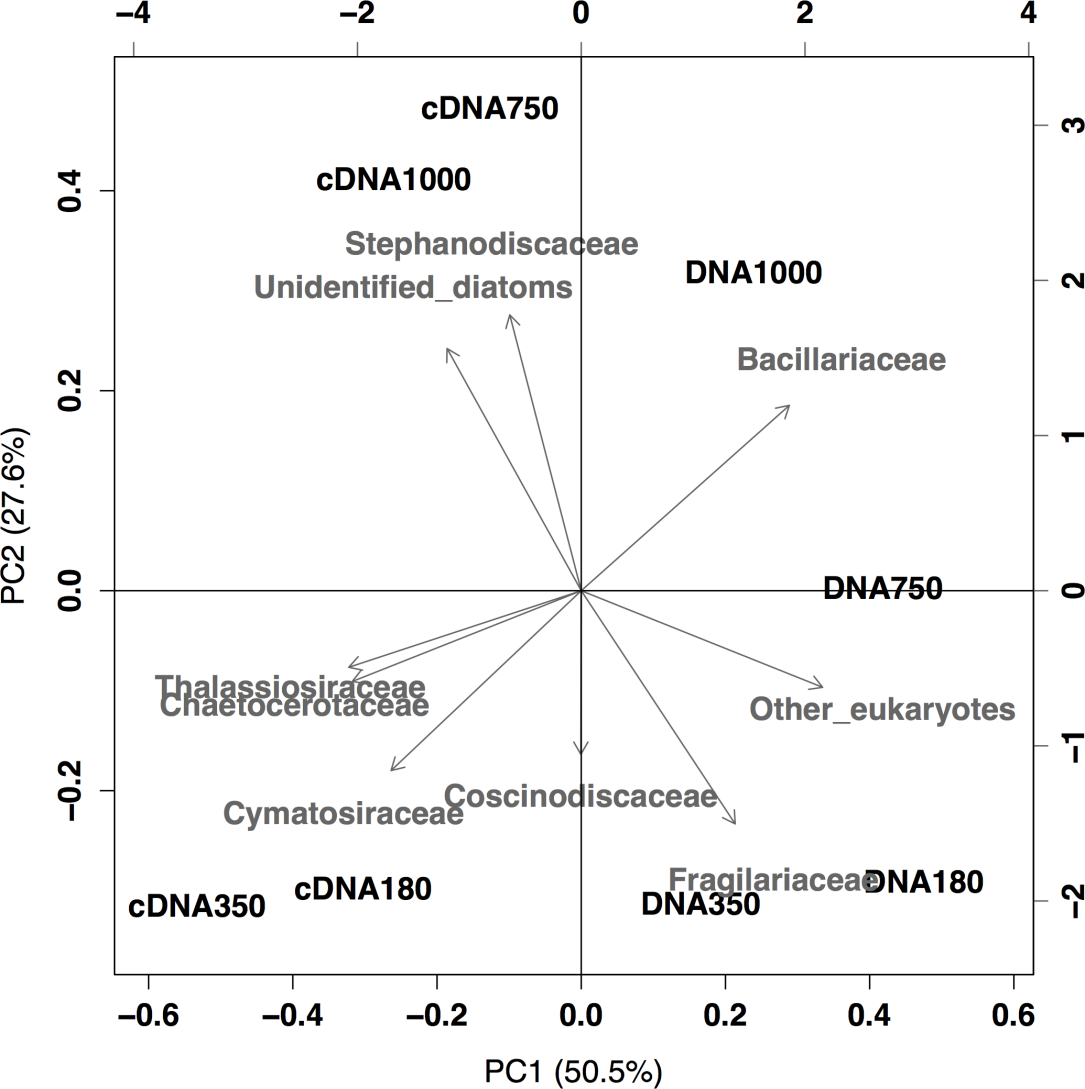
Principal component analysis (PCA) ordination plots incorporating relative contribution of sequence reads for *p*CO_2_ treatments in the DNA and cDNA libraries on day 3. Each sample is represented as the library type followed by the *p*CO_2_ treatments (i.e., DNA180 indicates the DNA sample from the 180 μatm CO_2_ treatment).

In the PCA plot (Fig 8), libraries were divided into DNA and cDNA by the first axis (PC1), and these were further divided to high (750 and 1000 μatm) and other (180 and 350 μatm) CO_2_ treatments by the second axis (PC2), except for the 750 μatm CO_2_ treatment in the DNA library. In the PCA analysis, the first and second axes from the ordination explained 50.5% and 27.6% of the total variability, respectively (cumulative contribution of 78.1%).

## Discussion

### 4.1. Chemical and biological characteristics of the incubation seawater

In our experiment, initial seawater *p*CO_2_ was intermediate value between pre- and post-bloom phases in the Oyashio region reported by Midorikawa et al. [20]. In addition, in the initial seawater, macronutrients were not depleted, and the Chl *a* concentration was lower than the values previously reported during the spring diatom blooms in this region (> 5 μg L^–1^, [20, 47, 60]). These results indicate that the intense diatom bloom has not yet occurred in the seawater used for the incubation experiment. This speculation is supported by our CHEMTAX analysis, which revealed that the initial phytoplankton assemblages were dominated by a mixture of diatoms and cryptophytes (Fig 3) as previously observed during the pre-bloom period [47, 60]. The initial value of *F*_v_/*F*_m_ was also similar to that in the pre-bloom phase in spring Oyashio water measured with fast repetition rate fluorometry (FRRF) by Yoshie et al. [61]. Concentrations of Chl *a* increased exponentially during the incubation (S2 Fig) and it was accompanied by the increases in diatom contribution to the Chl *a* biomass (Fig 3), suggesting that our incubation could simulate the early growth phase of the diatom bloom.

### 4.2. Effects of CO_2_ availability on the phytoplankton pigments and *F*_v_/*F*_m_

The net growth rates of Fuco were less in the low (180 μatm) and high (750 and 1000 μatm) CO_2_ treatments relative to the control (350 μatm), while no significant CO_2_ difference was observed in the other chemotaxonomic pigments (Fig 2), suggesting that the growth of diatoms could be selectively diminished by changes in CO_2_ availability. This assumption is supported by size-fractionated Chl *a* analysis, which showed that elevated CO_2_ levels selectively decreased large-sized (≥10 μm) phytoplankton cells, that were most likely composed of diatoms. Our results were inconsistent with the previous laboratory culture experiments, which indicated that diatoms tended to increase their growth under elevated CO_2_ levels [10, 11]. In addition, Wu et al. [62] demonstrated that the CO_2_ enrichment stimulated the growth of larger diatom species rather than that of smaller cells. However, field studies using natural phytoplankton assemblages showed positive [12, 13, 28] and negative [17, 27, 63] effects of increased CO_2_ availability on diatom growth. This pattern may have been caused by differences in diatom species [4, 19, 62], an effect of CO_2_ on zooplankton grazing [64], and/or the bioavailability of iron in response to the change in carbonate chemistry [18]. Our findings further support that the sensitivity of phytoplankton assemblages to elevated CO_2_ differs geographically, according to the differences in species composition and environmental conditions [5]. In the present study, decreased contributions of diatoms resulted in the relative increases in other phytoplankton taxa, such as cryptophytes and haptophytes (Fig 3). Our results suggest that CO_2_ enrichment may decrease the diatom contribution to the phytoplankton assemblages during the growth phase of spring blooms in the Oyashio region. However, it should be noted that our incubation period was short (i.e., 1–2 days under fully manipulated CO_2_ conditions) and may not have allowed for full acclimation of phytoplankton community to changes in CO_2_. Therefore, the biological responses observed in our study could be transient, and further work with a longer incubation period is also required to refine the results observed in our study.

Differences in *F*_v_/*F*_m_ values among treatments were not significant throughout the incubation (S3 Fig), indicating that CO_2_ availability had a minimal effect on the photochemical quantum efficiency of PSII for phytoplankton assemblages. Similar results were also obtained from Fe-fertilized field experiments [16, 18].

### 4.3. Effects of CO_2_ availability on the diatom-specific *rbcL* gene and its transcripts

The copy number of *rbcL* gene targeting diatoms exhibited a good correlation with Fuco concentration in this study (Fig 4). This indicates a close coupling between *rbcL* gene and the chemotaxonomic pigment in diatoms. A similar result was also observed during the CO_2_-controlled incubation experiment in the oceanic Bering Sea [27]. Given that Fuco can be a strong indicator of diatom carbon biomass in the Oyashio region [47], our result suggests that the diatom-specific *rbcL* gene could also serve as a potential indicator for diatom carbon biomass in the area.

The abundance of diatom *rbcL* gene transcripts decreased with an increase in *p*CO_2_ (Fig 5A), indicating that RubisCO expression could decrease in high CO_2_ environments. However, DNA-normalized *rbcL* transcription did not vary as a function of *p*CO_2_ level, indicating that the transcriptional activity was unaffected by CO_2_ availability. Pigment analysis by HPLC also indicated the decrease in diatom biomass under high CO_2_ conditions (Figs 2 and 3). Therefore, the decrease in *rbcL* transcript abundance in diatom community might be caused primarily by the decrease in diatom biomass rather than the decrease in transcriptional activity in diatom cells. A laboratory culture experiment using the diatom *Thalassiosira weissflogii* also showed a decrease in RubisCO concentration with an increase in CO_2_ concentration over a range of 182 to 750 ppm [65]. Decreases in *rbcL* transcripts or RubisCO concentrations under elevated CO_2_ conditions were also observed from the field incubation experiments conducted off the coast of California [65] and in the Bering Sea [27]. However, there is little evidence on CO_2_-induced transcriptional regulation of *rbcL* in diatom cells. Moreover, Losh et al. [65, 66] demonstrated that the growth and productivity of phytoplankton assemblages were not affected by increased CO_2_ level, although the RubisCO expression decreased. Their study suggested that the decrease in RubisCO content could be compensated by an increase in CO_2_ concentration around RubisCO at higher CO_2_ levels. In our study, therefore, the decrease in diatom growth rate under high CO_2_ levels might be unrelated to the transcriptional response of *rbcL* gene. One possible mechanism for the decrease in diatom biomass is that the increase in photoinhibition under elevated CO_2_ levels [62]. The shift in diatom community composition could also affect the abundance of diatom-specific *rbcL* and its transcripts (Fig 7).

Although our results cannot extrapolate to longer-term behaviors in the study area, short-term incubation may be suitable for determining physiological responses in the natural phytoplankton assemblages, because that can reduce artifacts derived from incubation (i.e., so-called bottle effects; [67, 68]). In addition, environmental parameters other than CO_2_ (e.g., nutrient availability) would be differentiated among CO_2_ treatments after longer incubation period [16, 69], and that complicated the source of the algal responses.

Our rarefaction curves reached plateaus with 10,000 reads for all samples (S5 Fig), suggesting that the sequencing efforts were sufficient to address the comparative analyses among libraries. Taxonomic analysis based on NGS revealed that community composition of *rbcL* gene and its transcripts differed largely among CO_2_ treatments on day 3. The result suggests that the diatom community structure shifted rapidly in response to changes in CO_2_ levels, whereas transcriptional activity of total diatom community was little affected by CO_2_ availability. In our NGS libraries, CO_2_-induced changes in the taxonomic composition of diatoms exhibited similar trends between DNA and cDNA. For example, the contributions of Chaetocerotaceae, Thalassiosiraceae, and Fragilariaceae consistently decreased in both the DNA and cDNA libraries in response to changes in CO_2_ levels compared to the control, whereas those of the Bacillariaceae increased (Fig 7). A possible explanation for the consistency is that the decrease and increase in *rbcL* transcription could be caused by the decrease and increase in *rbcL* gene abundance, respectively. Our results indicate that the effects of CO_2_ availability could differ among diatom taxa, resulting in changes in the community composition.

Elevated CO_2_ availability decreased the OTU richness and diversity of diatom-specific *rbcL* genes and their transcripts in our experiment (Table 3). Although the decreases in diversity indices were rather small, the diatom community lost 5–10% in terms of the *rbcL* OTU richness during the incubation. The PCA ordination revealed that the *rbcL* libraries were clearly separated by high and low CO_2_ treatments in both DNA and cDNA libraries (Fig 8), suggesting that the decreases in OTU diversity were accompanied with shifts in the diatom-specific *rbcL* composition.

In contrast to elevated CO_2_ treatments, the diversity, taxonomic composition, and transcription activity of diatom-specific *rbcL* were weakly affected by decreases in CO_2_ availability (Table 3, Figs 5 and 8), indicating that the diatom community in the study area was rather stable to low CO_2_ conditions compared with higher CO_2_ conditions. Previous field observations also showed that the diversity and productivity of diatom assemblages were maintained during the spring blooms in the Oyashio region [47, 61]. Our results suggest that the dominant diatom species during the spring blooms in this region could be acclimated to the bloom-induced low CO_2_ conditions every spring [20], whereas they may not acclimate well to a high CO_2_ environment that had not been experienced before. The acclimation would be accomplished by the use of CCMs mediated by the enzyme carbonic anhydrase (CA; [21, 22]). Burkhardt et al. [70] reported that the activity of CA increased in the low CO_2_ (36–180 μatm) treatments compared to the ambient (360 μatm) treatment. Furthermore, it has been proposed that bloom-forming diatom species possess highly efficient and tightly regulated CCMs to maintain their growth even under low CO_2_ environments [71]. However, decreases in the growth rate of Fuco and the number of *rbcL* OTUs observed in the low CO_2_ treatment, implying that some diatom clades could not respond to abrupt decrease in CO_2_ availability. Because there is inter-specific variation in the activity and plasticity in CCMs among diatoms [72, 73], one possible explanation of our results is that diatom species with low CCM capacity were eliminated in the low CO_2_ treatments.

## Conclusions

In the present study, we focused on the effects of different CO_2_ availability on the growth, community composition, and photosynthetic capacity of diatoms in the spring Oyashio waters of the western North Pacific. The comprehensive NGS analyses for the diatom-specific *rbcL* gene and its transcripts enabled us, for the first time, to detect significant changes in taxonomic composition and diversity of diatoms among CO_2_ treatments even under the short-term incubation. Consequently, we revealed that the elevated CO_2_ availability decreased the OTU richness and diversity of diatom-specific *rbcL* gene and their transcripts. Interestingly, the increase in seawater CO_2_ levels reduced the growth rate of diatoms and their relative contribution to the Chl *a* biomass as estimated from photosynthetic pigment signatures. In addition, the transcript abundance of diatom-specific *rbcL* gene also decreased under elevated CO_2_ levels. According to Chiba et al. [74], a decrease in springtime Chl *a* concentration accompanied the changes in the predominant diatoms from centrics to pennates during the 1960s and 1990s in the Oyashio region. Additionally, the bloom dynamics could be controlled by physical factors such as the Pacific Decadal Oscillation (PDO, [75]). Therefore, it is crucial to investigate the relationship between environmental changes in Oyashio waters and the dynamics and mechanisms of its spring diatom blooms. We believe that molecular biological techniques can help us to gain a deeper understanding of the changes in the composition and physiology of spring Oyashio diatoms.

## Supporting Information

S1 Fig Temporal changes in (a) *p*CO_2_ and (b) pH. Error bars denote ± 1 SD (n = 2 or 3).
(EPS)

S2 Fig Temporal changes in the concentrations of Chl *a*. Error bars denote ± 1 SD (n = 3).
(EPS)

S3 Fig Temporal changes in *F*_v_/*F*_m_. Error bars denote ± 1 SD (n = 3).
(EPS)

S4 Fig Neighbor-joining tree based on partial *rbcL* gene reference sequences used for the taxonomic classification. Taxonomic groups are distinguished by different symbols in the tree. Reference sequences which could not be identified as a specific diatom family (i.e., a contig sequence was most closely related to two or more reference sequences that belong to different family) are shown as the gray circle.
(TIFF)

S5 Fig Rarefaction analysis of the diatom-specific *rbcL* (a) DNA and (b) cDNA libraries of the initial sample and the 180, 350, 750, and 1000 μatm CO_2_ treatments on day 3. The rarefaction curves, plotting the number of operational taxonomic units (OTUs) as a function of the number of sequences, were computed by the mothur software package.
(EPS)

S1 Table Initial pigment:Chl *a* ratios for CHEMTAX analysis: (A) True ratio matrix of Suzuki et al. (2002); (B) double and (C) half the ratios of (A); (D) assigned ratios of 0.75, 0.50 and 0.25 to each element following the method of Latasa (2007).
(DOCX)

S2 Table Final pigment:Chl *a* ratio matrices obtained by CHEMTAX program. (DOCX)

S3 Table Quality statistics of pre- and post-quality control sequence data. Sequence data were obtained from DNA and cDNA samples collected on days 0 and 3.
(DOCX)
