# Supplementary figures and images for: Response of Spring Diatoms to CO2 Availability in the Western North Pacific as Determined by Next-Generation Sequencing
Source: PLoS One. 2016 Apr 28;11(4):e0154291. doi: 10.1371/journal.pone.0154291 (PMC4849754; doi:10.1371/journal.pone.0154291)

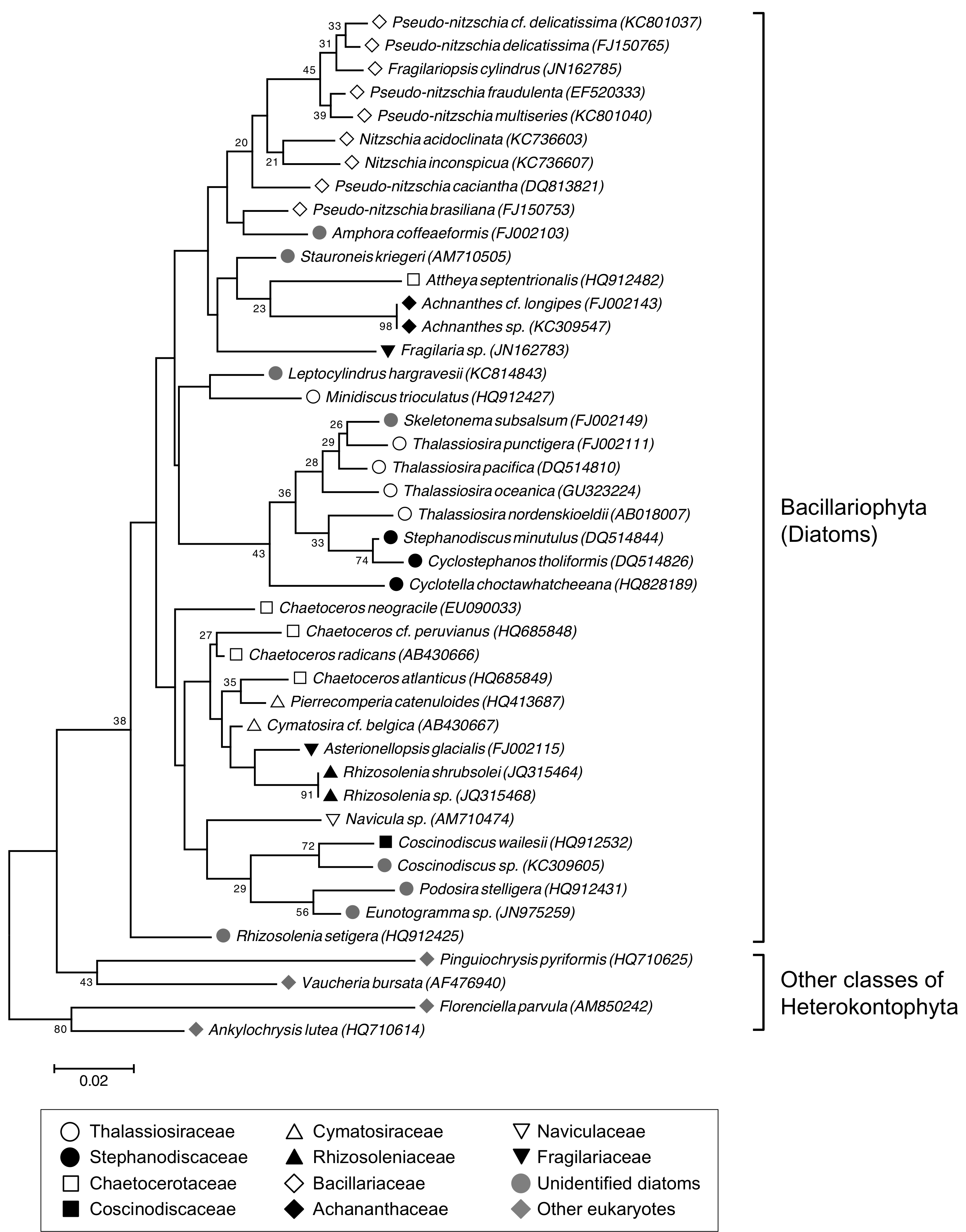

Supplement: S4 Fig — Taxonomic groups are distinguished by different symbols in the tree. Reference sequences which could not be identified as a specific diatom family (i.e., a contig sequence was most closely related to two or more reference sequences that belong to different family) are shown as the gray circle. (TIFF) [file pone.0154291.s004.tiff]
