# Supplementary material for: Response of Spring Diatoms to CO2 Availability in the Western North Pacific as Determined by Next-Generation Sequencing
Source: PLoS One. 2016 Apr 28;11(4):e0154291. doi: 10.1371/journal.pone.0154291 (PMC4849754; doi:10.1371/journal.pone.0154291)
Supplement: S1 Table — Initial pigment:Chl a ratios for CHEMTAX analysis: (A) True ratio matrix of Suzuki et al. (2002); (B) double and (C) half the ratios of (A); (D) assigned ratios of 0.75, 0.50 and 0.25 to each element following the method of Latasa (2007). (DOCX) [file pone.0154291.s006.docx]

**S1 Table.** Initial pigment:Chl *a* ratios for CHEMTAX analysis: (A) True ratio matrix of Suzuki et al. (2002); (B) double and (C) half the ratios of (A); (D) assigned ratios of 0.75, 0.50 and 0.25 to each element following the method of Latasa (2007).

|  | Fuco | 19'-But | 19'-Hex | Peri | Diadinox | Allo | Violax | Prasinox | Chl *b* | Zeax | Chl *a* |
| --- | --- | --- | --- | --- | --- | --- | --- | --- | --- | --- | --- |
| (A) |  |  |  |  |  |  |  |  |  |  |  |
| Diatoms | 0.75 | 0 | 0 | 0 | 0.24 | 0 | 0 | 0 | 0 | 0 | 1 |
| Hapto | 0 | 0 | 1.4 | 0 | 0.16 | 0 | 0 | 0 | 0 | 0 | 1 |
| Pelago | 0.62 | 0.93 | 0 | 0 | 0.44 | 0 | 0 | 0 | 0 | 0 | 1 |
| Chloro | 0 | 0 | 0 | 0 | 0 | 0 | 0.03 | 0 | 0.28 | 0.06 | 1 |
| Prasino | 0 | 0 | 0 | 0 | 0 | 0 | 0.11 | 0.36 | 0.89 | 0 | 1 |
| Crypto | 0 | 0 | 0 | 0 | 0 | 0.14 | 0 | 0 | 0 | 0 | 1 |
| Dino | 0 | 0 | 0 | 0.53 | 0 | 0 | 0 | 0 | 0 | 0 | 1 |
| Cyano | 0 | 0 | 0 | 0 | 0 | 0 | 0 | 0 | 0 | 0.33 | 1 |
|  |  |  |  |  |  |  |  |  |  |  |  |
| (B) |  |  |  |  |  |  |  |  |  |  |  |
| Diatoms | 1.5 | 0 | 0 | 0 | 0.48 | 0 | 0 | 0 | 0 | 0 | 1 |
| Hapto | 0 | 0 | 2.8 | 0 | 0.32 | 0 | 0 | 0 | 0 | 0 | 1 |
| Pelago | 1.24 | 1.86 | 0 | 0 | 0.88 | 0 | 0 | 0 | 0 | 0 | 1 |
| Chloro | 0 | 0 | 0 | 0 | 0 | 0 | 0.06 | 0 | 0.56 | 0.12 | 1 |
| Prasino | 0 | 0 | 0 | 0 | 0 | 0 | 0.22 | 0.72 | 1.78 | 0 | 1 |
| Crypto | 0 | 0 | 0 | 0 | 0 | 0.28 | 0 | 0 | 0 | 0 | 1 |
| Dino | 0 | 0 | 0 | 1.06 | 0 | 0 | 0 | 0 | 0 | 0 | 1 |
| Cyano | 0 | 0 | 0 | 0 | 0 | 0 | 0 | 0 | 0 | 0.66 | 1 |
|  |  |  |  |  |  |  |  |  |  |  |  |
| (C) |  |  |  |  |  |  |  |  |  |  |  |
| Diatoms | 0.375 | 0 | 0 | 0 | 0.12 | 0 | 0 | 0 | 0 | 0 | 1 |
| Hapto | 0 | 0 | 0.7 | 0 | 0.08 | 0 | 0 | 0 | 0 | 0 | 1 |
| Pelago | 0.31 | 0.465 | 0 | 0 | 0.22 | 0 | 0 | 0 | 0 | 0 | 1 |
| Chloro | 0 | 0 | 0 | 0 | 0 | 0 | 0.015 | 0 | 0.14 | 0.03 | 1 |
| Prasino | 0 | 0 | 0 | 0 | 0 | 0 | 0.055 | 0.18 | 0.445 | 0 | 1 |
| Crypto | 0 | 0 | 0 | 0 | 0 | 0.07 | 0 | 0 | 0 | 0 | 1 |
| Dino | 0 | 0 | 0 | 0.265 | 0 | 0 | 0 | 0 | 0 | 0 | 1 |
| Cyano | 0 | 0 | 0 | 0 | 0 | 0 | 0 | 0 | 0 | 0.165 | 1 |
|  |  |  |  |  |  |  |  |  |  |  |  |
| (D) |  |  |  |  |  |  |  |  |  |  |  |
| Diatoms | 0.75 | 0 | 0 | 0 | 0.25 | 0 | 0 | 0 | 0 | 0 | 1 |
| Hapto | 0 | 0 | 0.75 | 0 | 0.25 | 0 | 0 | 0 | 0 | 0 | 1 |
| Pelago | 0.75 | 0.75 | 0 | 0 | 0.5 | 0 | 0 | 0 | 0 | 0 | 1 |
| Chloro | 0 | 0 | 0 | 0 | 0 | 0 | 0.25 | 0 | 0.5 | 0.25 | 1 |
| Prasino | 0 | 0 | 0 | 0 | 0 | 0 | 0.25 | 0.5 | 0.75 | 0 | 1 |
| Crypto | 0 | 0 | 0 | 0 | 0 | 0.25 | 0 | 0 | 0 | 0 | 1 |
| Dino | 0 | 0 | 0 | 0.5 | 0 | 0 | 0 | 0 | 0 | 0 | 1 |
| Cyano | 0 | 0 | 0 | 0 | 0 | 0 | 0 | 0 | 0 | 0.5 | 1 |

Abbreviations: Hapto, Haptophytes; Pelago, Pelagophytes; Chloro, Chlorophytes; Crypto, Cryptophytes; Dino, Dinoflagellates; Cyano, Cyanobacteria; Fuco, Fucoxanthin; 19’-But, 19’-Butanoyloxyfucoxanthin; 19’-Hex, 19’-Hexanoyloxyfucoxanthin; Peri, Peridinin, Diadinox, Diadinoxanthin; Allo, Alloxanthin; Violax, Violaxanthin; Prasinox, Prasinoxanthin; Chl *b*, Chlorophyll *b*; Zeax, Zeaxanthin; Chl *a*, Chlorophyll *a*.

**Additional references not found in the text**

1. Suzuki K, Minami C, Liu H, Saino T. Temporal and spatial patterns of chemotaxonomic algal pigments in the subarc- tic Pacific and the Bering Sea during the early summer of 1999. Deep-Sea Res. II. 2002; 49: 5685–5704.
2. Latasa M. Improving estimations of phytoplankton class abundances using CHEMTAX. Mar. Ecol. Prog. Ser. 2007; 329: 13–21.
