# Supplementary material for: Response of Spring Diatoms to CO2 Availability in the Western North Pacific as Determined by Next-Generation Sequencing
Source: PLoS One. 2016 Apr 28;11(4):e0154291. doi: 10.1371/journal.pone.0154291 (PMC4849754; doi:10.1371/journal.pone.0154291)
Supplement: S2 Table — (DOCX) [file pone.0154291.s007.docx]

**S2 Table.** Final pigment:Chl *a* ratio matrices obtained by CHEMTAX program.

|  | Fuco | 19'-But | 19'-Hex | Peri | Diadinox | Allo | Violax | Prasinox | Chl *b* | Zeax | Chl *a* |
| --- | --- | --- | --- | --- | --- | --- | --- | --- | --- | --- | --- |
|  |  |  |  |  |  |  |  |  |  |  |  |
| Diatoms | 0.695 | 0 | 0 | 0 | 0.154 | 0 | 0 | 0 | 0 | 0 | 1 |
| Hapto | 0 | 0 | 1.001 | 0 | 0.137 | 0 | 0 | 0 | 0 | 0 | 1 |
| Pelago | 0.567 | 0.725 | 0 | 0 | 0.479 | 0 | 0 | 0 | 0 | 0 | 1 |
| Chloro | 0 | 0 | 0 | 0 | 0 | 0 | 0.007 | 0 | 0.292 | 0.047 | 1 |
| Prasino | 0 | 0 | 0 | 0 | 0 | 0 | 0.153 | 0.369 | 1.008 | 0 | 1 |
| Crypto | 0 | 0 | 0 | 0 | 0 | 0.147 | 0 | 0 | 0 | 0 | 1 |
| Dino | 0 | 0 | 0 | 0.618 | 0 | 0 | 0 | 0 | 0 | 0 | 1 |
| Cyano | 0 | 0 | 0 | 0 | 0 | 0 | 0 | 0 | 0 | 0.332 | 1 |

Abbreviations: as in Table S1.
