# Supplementary material for: Response of Spring Diatoms to CO2 Availability in the Western North Pacific as Determined by Next-Generation Sequencing
Source: PLoS One. 2016 Apr 28;11(4):e0154291. doi: 10.1371/journal.pone.0154291 (PMC4849754; doi:10.1371/journal.pone.0154291)
Supplement: S3 Table — Sequence data were obtained from DNA and cDNA samples collected on days 0 and 3. (DOCX) [file pone.0154291.s008.docx]

**S3 Table. Quality statistics of pre- and post-quality control sequence data**.

|  |  | Raw reads | | |  | Quality-filtered reads | | |
| --- | --- | --- | --- | --- | --- | --- | --- | --- |
|  |  | Number of reads | Mean read length | Mean quality score |  | Number of reads | Mean read length | Mean quality score |
| DNA | Initial | 1,135,818 | 119.24 | 28.27 |  | 291,029 | 112.90 | 31.22 |
|  | 180 μatm | 1,123,203 | 114.50 | 27.99 |  | 280,358 | 112.92 | 31.29 |
|  | 350 μatm | 1,194,481 | 112.65 | 27.80 |  | 269,197 | 112.89 | 31.22 |
|  | 750 μatm | 1,118,214 | 117.31 | 28.09 |  | 275,419 | 112.89 | 31.16 |
|  | 1000 μatm | 1,075,315 | 113.31 | 27.88 |  | 248,598 | 112.90 | 31.20 |
| cDNA | Initial | 340,809 | 114.05 | 27.75 |  | 66,002 | 112.97 | 31.51 |
|  | 180 μatm | 588,581 | 105.15 | 27.16 |  | 98,083 | 112.97 | 31.57 |
|  | 350 μatm | 616,230 | 104.06 | 27.07 |  | 99,429 | 113.11 | 31.54 |
|  | 750 μatm | 533,061 | 107.33 | 27.33 |  | 90,303 | 112.97 | 31.51 |
|  | 1000 μatm | 447,990 | 107.63 | 27.33 |  | 78,289 | 112.97 | 31.52 |
